# Endothelial progenitor cells for diabetic cardiac and kidney disease

**DOI:** 10.1093/stcltm/szae025

**Published:** 2024-05-11

**Authors:** Matthew J Raleigh, Sachin V Pasricha, Aaron Nauth, Michael R Ward, Kim A Connelly

**Affiliations:** Keenan Research Centre for Biomedical Science, St. Michael’s Hospital, Unity Health, Toronto, Ontario M5B 1T8, Canada; Division of Orthopaedic Surgery, Department of Surgery, University of Toronto, Toronto, Ontario M5T 1P5, Canada; Division of Nephrology, Department of Medicine, University of Toronto, Toronto, Ontario M5G 1V7, Canada; Division of Orthopaedic Surgery, Department of Surgery, University of Toronto, Toronto, Ontario M5T 1P5, Canada; London Health Sciences Centre, London, Ontario N6A 5W9, Canada; Division of Cardiology, Department of Medicine, University of Western Ontario, London, Ontario N6A 5A5, Canada; Keenan Research Centre for Biomedical Science, St. Michael’s Hospital, Unity Health, Toronto, Ontario M5B 1T8, Canada; Divison of Cardiology, Department of Medicine, University of Toronto, Toronto, Ontario M5S 3H2, Canada

**Keywords:** diabetes, cardiac, kidney, stem/progenitor cells, tissue regeneration

## Abstract

The management of diabetes mellitus and its resultant end organ dysfunction represents a major challenge to global health-care systems. Diabetic cardiac and kidney disease commonly co-occur and are significant contributors to the morbidity and mortality of patients with diabetes, carrying a poor prognosis. The tight link of these parallel end organ manifestations suggests a deeper common underlying pathology. Here, we outline the mechanistic link between diabetic cardiac and kidney disease, providing evidence for the role of endothelial dysfunction in both processes and the potential for cellular therapy to correct these disorders. Specifically, we review the preclinical and clinical evidence for endothelial progenitor cell therapy in cardiac, kidney, and cardio-renal disease applications. Finally, we outline novel approaches to endothelial progenitor cell therapy through cell enhancement and the use of extracellular vesicles, discussing published and future work.

**Significance Statement**

This review links diabetic cardiac and kidney disease from the clinical to the cellular level, providing an understanding of the common processes, and thus common opportunities for intervention. Furthermore, this article describes a potential common cellular therapy, outlining the preclinical and clinical evidence, while highlighting the issues and potential next steps. Thus, this review provides a framework for planning future investigations and therapies for these disease processes.

## Introduction

An estimated 536.6 million people had diabetes mellitus (DM) as of 2021.^1^ Collectively, these individuals accounted for an associated healthcare expenditure of $966 billion (all figures in USD) in 2021.^1^ Alarmingly, the prevalence of DM is still increasing, with 783.2 million people projected to be diagnosed with DM by 2045, leading to an estimated annual healthcare expenditure of $1.05 trillion.^1^

Therefore, understanding the pathogenesis of DM and preventing its micro- and macro-vascular complications is a global healthcare priority. Complications of particular interest are diabetic kidney (DKD) and diabetic cardiovascular disease (CVD), (formerly thought of as solely atherosclerotic coronary artery disease), given their significant prevalence, cost, and mortality.^2^

DKD develops in nearly half of all individuals with type 2 diabetes.^3^ Further, roughly 30% of diabetics worldwide develop end-stage kidney disease necessitating dialysis or kidney transplantation,^4^ with the cost of DKD for those on dialysis estimated to be $21 900 per month.^5^ Diabetic CVD has a similar prevalence, impacting 32% of diabetics worldwide.^6^ Likewise, the addition of CVD increases the cost of diabetes care by $3500-$10 000.^7^ Beyond their high prevalence and cost, DKD and diabetic CVD significantly impact mortality. In fact, cardiovascular disease, kidney disease, and diabetes account for nearly 40% of deaths in developed nations.^8^

Moreover, cardiovascular and kidney disease often occur concurrently in patients with DM, with more than half of diabetics over 75 years of age having both entities,^9^ which is associated with worse a prognosis for patients.^10^ More importantly, this concurrence and synergistic interaction imply a common pathogenesis, and by extension, potentially common interventions.

This review provides the evidence for endothelial dysfunction as a driver of diabetic kidney and cardiovascular disease and outlines the relevant preclinical and clinical studies investigating the application of cellular therapy for the management of these intertwined pathologies.

## Endothelial dysfunction in diabetic cardiac and kidney disease

One of the common mechanisms of injury in diabetic cardiac and kidney disease is endothelial dysregulation.^11^ Indeed, this is a cardinal feature of DM, manifesting on both the macro- and micro-vascular levels,^12^ and is implicated in the development of diabetic cardiac and kidney disease. Reduced arterial reactivity has been demonstrated in diabetics,^13^ with such changes implicated in CVD^14^ and linked to its progression.^15,16^ Similarly, endothelial dysfunction is highly associated with kidney disease.^17,18^ Reduced flow-mediated-vasodilation has been found in patients with end-stage kidney disease on dialysis,^19,20^ but is also present in patients with early-stage disease (ie, stages II–III).^21^ Indeed, the presence of detectable reductions in vascular responsiveness at relatively early stages of disease highlights the relationship between a dysregulated endothelium and kidney disease. Evidently, then, endothelial dysfunction plays a key role in the development of diabetic cardiovascular and kidney disease.

The mechanisms underlying endothelial dysfunction in diabetes have been thoroughly investigated through preclinical and clinical investigations. Hyperglycemia alone can result in vasodilatory dysfunction acutely in healthy subjects^22,23^ and chronically in type II diabetics.^24^ Such findings reflect deleterious changes at the cellular level, as hyperglycemia induces pathological shifts in metabolism. Hyperglycemia inhibits glucose-6-phosphate dehydrogenase,^25^ reducing activity through the pentose phosphate pathway, thereby shifting metabolism toward the polyol and the hexosamine pathway,^26^ with reciprocal reduction in glycolytic pathways.^27^ Such metabolic shifts result in reduced nicotinamide adenine dinucleotide phosphate (NADPH) availability, limiting antioxidant activity.^28^ Simultaneously, increased activity in the polyol pathway generates 3-deoxyglucosone, ultimately forming advanced glycation end products (AGEs).^29^ Thus, 2 key consequences of hyperglycemia are an increase in reactive oxygen species (ROS) and the formation of AGEs.

Increases in ROS and AGEs further disrupt normal cellular metabolism and signaling, alter function, and induce pathology.^27^ Foremost among these disruptions is the reduced availability of nitric oxide (NO), a key signaling molecule that regulates vascular tone and exerts significant paracrine effects. NO availability is reduced through nitric oxide synthase inhibition via the described metabolic shifts of hyperglycemia,^30,31^ and by consumption through reaction with ROS. Furthermore, AGEs associate with intra- and extra-cellular proteins, altering their function. AGEs interact with cell-associated binding proteins, which signal the production of factors such as interleukin-1β, tissue necrosis factor-α, tissue growth factor-β (TGF-β), and platelet-derived growth factor in macrophages and mesangial cells, as well as vascular cell adhesion molecule-1 and thrombomodulin in endothelial cells (ECs).^32^ Moreover, the increase in ROS furthers pro-inflammatory signaling and increases oxidative stress in ECs. Such excessive oxidative stress can result in EC senescence and apoptosis,^33-35^ with alterations in cell-extracellular matrix interactions decreasing EC adhesion,^36^ correlating with the increased ECs in circulation and the EC loss noted in diabetics.^36-38^ Thus, diabetic hyperglycemia initiates both acute and chronic changes to the endothelial cell, surrounding cells, and extracellular matrix, inducing the changes collectively referred to as diabetic endothelial dysfunction.

Such endothelial dysfunction underlies the development of DKD and is detectable prior to overt manifestations of DKD.^39^ The glomerulus is sensitive to such changes as the relationship among ECs, basement membrane, and podocytes regulate the glomerular ultrafiltrate.^40^ Hyperglycemia and EC dysfunction generate a proinflammatory environment initiating pathological signaling between ECs and podocytes.^41^ In such an environment, podocytes overproduce vascular endothelial growth factor which, in the diabetic milieu, increases oxidative stress in ECs and podocytes.^42,43^ Further, hyperglycemia-induced secretion of TGF-β exerts negative effects on podocytes and ECs and stimulates podocyte production of endothelin-1.^41^ Endothelin-1 increases ROS in ECs while decreasing the thickness of the endothelial glycocalyx.^44^ Other pro-inflammatory molecules also propagate endothelial dysfunction—namely chemokine (C-X-C motif) ligand 1—which promotes a hyperglycemic environment and peritoneal membrane endothelial dysfunction in diabetics, ultimately limiting the duration of peritoneal dialysis as a viable therapy.^45^ Indeed, the clearing of these pro-inflammatory toxins, also called “middle molecules,” is the target for expanded hemodialysis techniques such as hemodiafiltration.^46^ Altogether, the pro-inflammatory processes that result from hyperglycemia disrupt the basement membrane and induce podocyte hypertrophy, dropout, and apoptosis, ultimately resulting in glomerular dysfunction and DKD.

Likewise, hyperglycemic endothelial dysfunction significantly contributes to the development of diabetic CVD. While the association with atherosclerosis, and by extension, coronary artery disease, is a well-established result of the endothelial dysfunction noted above, the endothelium also plays a major role in the development of diabetic cardiomyopathy. This is especially true as it relates to the development of heart failure with preserved ejection fraction, which is highly associated with DM.^47^ As initially outlined by Paulus and Tschöpe,^48^ the dysfunction seen in ECs in a diabetic environment (ie, increased ROS, reduced NO) results in cardiomyocyte hypertrophy and rigidity, as well as subendothelial invasion by monocytes, stimulating fibroblasts, and increasing collagen deposition. In contrast, heart failure with reduced ejection fraction occurs due to a loss of functional cardiomyocytes from apoptosis and necrosis, with resultant fibrotic changes, stemming from chronic ischemia, or infarction.^49^ Such ischemia results, however, from the described endothelial dysfunction and injury in DM. Clearly, then, the endothelium is one of the key drivers of diabetic cardiomyopathy and CVD.

**Figure 1.**
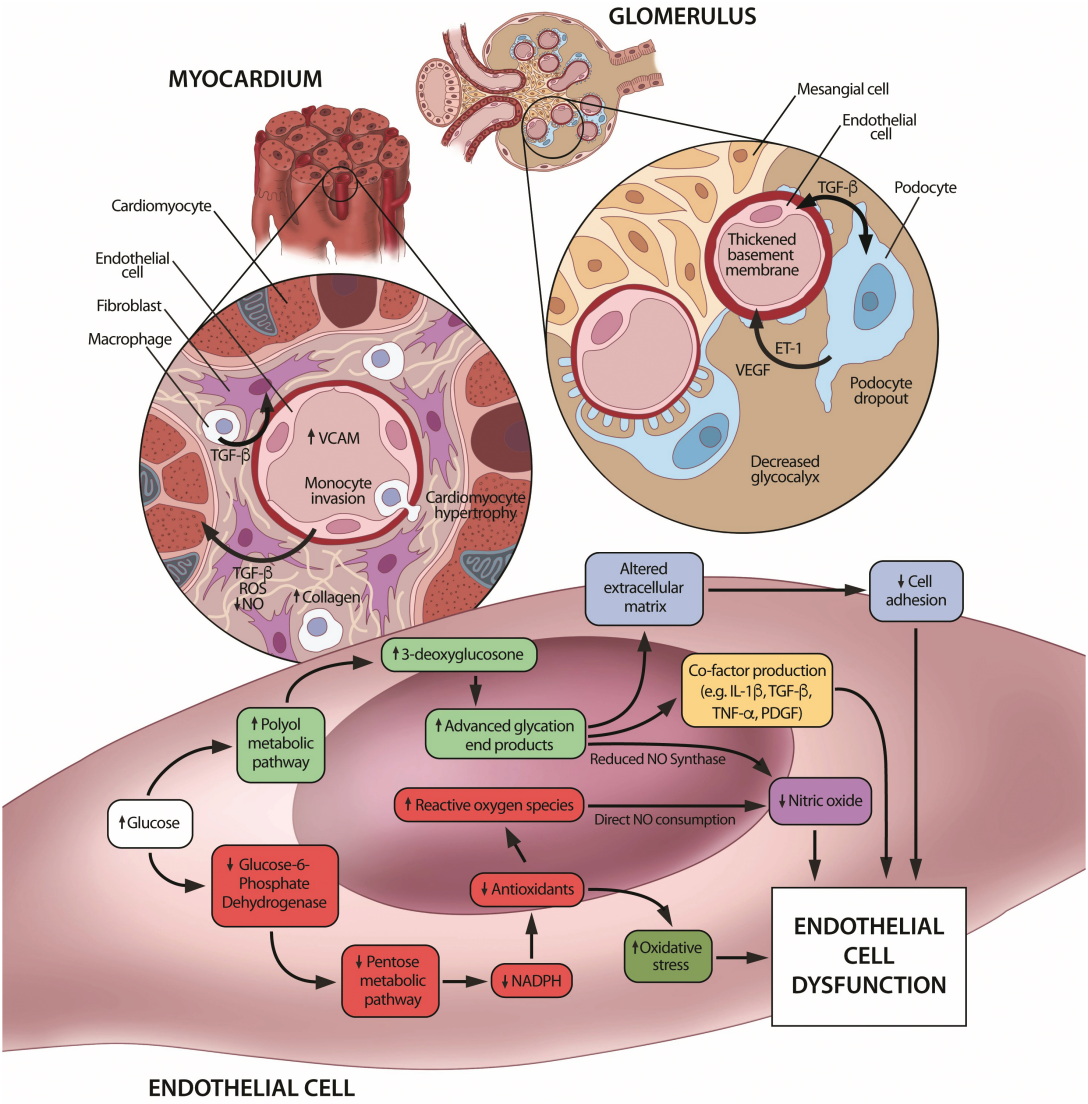
Diagram depicting the common endothelial dysfunction in diabetic cardiac and kidney disease. The molecular mechanisms driving the cellular dysfunction underlying the end organ manifestations are depicted (central endothelial cell). Pathological changes in the local tissues and surrounding cells secondary to this endothelial cell dysfunction in both the heart and kidney at the level of the myocardium and glomerulus are shown (left and right sides respectively). In the heart, endothelial cells drive the pathology seen in diabetic cardiac disease (ie, heart failure with preserved ejection fraction). Here, paracrine signaling from the endothelial cells induce cardiomyocyte hypertrophy and collagen deposition by fibroblast activation and monocyte invasion. Likewise, in the kidney endothelial cells within the renal corpuscle engage in reciprocal signaling with podocytes, inducing the loss of podocyte foot processes, their hypertrophy and subsequent dropout. Simultaneous alterations of the surrounding tissues by thickening of the basement membrane and decreasing of the glycocalyx occurs. Such changes culminate in altered barrier function, albuminuria, and diabetic kidney disease. Abbreviations: ROS, reactive oxygen species, NO, nitric oxide, VCAM, vascular cell adhesion molecule, TGF-β, tissue growth factor β, TNF-α, tissue necrosis factor α, ET-1, endothelin 1, VEGF, vascular endothelial growth factor, NADPH, nicotinamide adenine dinucleotide phosphate, IL-1β, interleukin 1β.

Thus, endothelial dysfunction, stemming from endothelial cell injury, is a feature of diabetes and is a key driver in the related organ dysfunction, with clear mechanistic links to diabetic cardiac and kidney disease (see Figure 1 for a summary of mechanisms).

## Endothelial progenitor cells in diabetes, cardiovascular disease, and kidney disease

Given the role and significant clinical implications of endothelial dysfunction in diabetes, the management and reversal of this dysfunction are critical in the prevention of diabetic end organ pathology. As discussed, hyperglycemia injures the EC, initiating a negative cascade of paracrine signaling and endothelial dysfunction. Endothelial repair, however, is limited and requires appropriately functioning local ECs within a homeostatic milieu,^50^ which is absent in DM.^51^ Therefore, extrinsic repair and regenerative approaches are key in addressing the EC dysfunction and loss in diabetes.^37^

Endothelial progenitor cells (EPCs) are a population of bone marrow-derived cells that can facilitate endothelial repair and reverse dysfunction. As originally described in 1997 by Asahara et al, EPCs were derived from mononuclear cells (MNC) and were felt to represent hemangioblasts based on their co-expression of hematopoietic and endothelial cell markers, in vitro appearance, and in vivo activity.^52^ Since their initial discovery, many descriptions have been proposed to identify a specific cell population with endothelial regenerative capacity. This includes the co-expression of CD34 and CD133 with vascular endothelial growth factor receptor 2, CD31, E-selectin, or CD105.^53^

However, a canonical expression pattern remained difficult to establish, and so a functional assessment was used. This approach identified populations of cells by their relative proliferative capacity, lineage differentiation, and contribution to vessel formation. From this framework, 2 populations of cells have been identified. These are respectively, myeloid angiogenic cells, previously known as “early EPCs,” and endothelial colony-forming cells, previously referred to as “late EPCs.”^53^ Endothelial colony-forming cells are clonogenic, more closely resemble an endothelial cell, and mediate regeneration by direct vascular integration or formation and by paracrine signaling.^53-56^ In contrast, myeloid angiogenic cells are non-clonogenic, resemble cells of the myeloid lineage, and mediate their regenerative effects via potent paracrine signaling, but do not directly participate in vessel formation.^53-57^

Despite such differences, both populations have been effective in preclinical models of vascular disease, including ischemic hindlimb models, diabetic retinopathy, and myocardial infarction.^58-61^ Thus, while differences exist in mechanism of action, the regenerative capacity of EPCs of various definitions remains well documented and supported in vivo. Accordingly, we will use the term EPC here to refer to a population of cells derived from the MNC population, maintained under endothelial cell culture conditions, which jointly express hematopoietic stem cells and endothelial cell makers. Though imprecise, given the inclusion of both myeloid angiogenic cells and endothelial colony-forming cells, this categorization encompasses the regenerative cell population outlined in the bulk of the existing literature.

Many clinical investigations have demonstrated correlations between disease states and EPC number and function. In patients with DM, EPC number and function are reduced in relation to disease progression.^62,63^ Correspondingly, circulating EPC numbers track with worsening vascular status in diabetics^64^ and are predictive of microvascular complications.^65^ Moreover, EPCs from diabetic patients have reduced functional and regenerative capacity,^66,67^ correlating with worsening diabetic comorbidities.^68^ Likewise, circulating EPC levels negatively correlate with CVD burden,^69^ can be used to predict future cardiovascular events,^70^ and demonstrate functional defects.^71^ Similarly, patients with kidney failure demonstrate reductions in EPC numbers and their regenerative capacity,^72,73^ with EPC numbers correlating to the degree of kidney dysfunction.^74^

Overall, EPCs represent an important potential therapy for endothelial dysfunction given the limited degree of intrinsic endothelial repair and the reductions in EPC number and function with the progression of diabetes, cardiovascular, and kidney disease.

## Preclinical applications of EPCs for cardiac and kidney disease

In support of these correlations, multiple preclinical investigations have demonstrated the effectiveness of EPC therapy in both acute and chronic models of cardiac and kidney disease.

### EPCs in cardiac disease models

Explorations of the potency of EPC therapy in models of cardiac disease were performed as an extension of early work demonstrating the potent angiogenic activity of EPCs in models of acute limb ischemia.^58-61^ Analogously, investigators evaluated the potential of EPCs to ameliorate cardiac function and angiogenesis in models of myocardial infarction.

Early investigations by Kawamoto et al^75^ and Kocher et al^76^ outlined the effectiveness of intravenously delivered human EPCs following left anterior descending artery ligation in immunodeficient rats. These studies demonstrated human EPC localization to the peri-infarct region and local microvasculature, with significant improvements in ventricular function and fibrosis. Expanding on this, Kim et al^77^ demonstrated similar benefits with intramyocardial delivery of human blood-derived EPCs. Such EPC therapy appears to function in a dose-dependent fashion, with an approximate doubling in capillary density, improvements in left ventricle (LV) function, and reduction of fibrosis with high (1 × 10^5^) compared to low (1 × 10^3^) dose EPC therapy.^78^

While these studies demonstrate the effectiveness of EPC therapy in an acute model of cardiac disease, EPCs have also been shown to be effective in models of chronic cardiac disease. By employing an ameroid constrictor to the proximal left anterior descending artery, a model of chronic myocardial ischemia can be generated, mimicking the chronic cardiac ischemia that occurs with endothelial dysfunction. Both Fuchs and Kawamoto et al used this technique to evaluate the potency of bone marrow MNC-expressing endothelial markers in pigs.^79,80^ In both studies, cell therapy delivered locally to the ischemic myocardium weeks following initiation of ischemia not only prevented deterioration but also improved LV function compared to the control animals, with corresponding histologic improvements. Such models more accurately depict the chronic and progressive nature of diabetic cardiac disease and highlight the potential of EPC therapy for this application.

### EPCs in kidney disease models

As with cardiac disease, laboratory studies have been performed to evaluate the effectiveness of EPCs for the treatment of kidney disease. In models of acute kidney injury secondary to ischemia, EPC delivery both systemically,^81-84^ or locally via injection to the subcapsular space,^85^ significantly reduces resultant interstitial fibrosis, tubular necrosis, and apoptosis, as well as post-injury endothelial to mesenchymal cell transition.^81^ Similar effects were observed in a model of glomerulonephritis.^86^ Here, mesangial expansion, macrophage invasion, and alpha-smooth muscle actin expression were all significantly reduced 1 week following EPC treatment, although proteinuria was not corrected. This was potentially due to the early time point of investigation compared to other studies employing an acute kidney injury model (1 vs 4 + weeks).

Likewise, EPCs demonstrate significant therapeutic benefits in models of chronic kidney disease (CKD). In a model of CKD induced by subtotal bilateral nephrectomy in rodents, a single intra-vascular delivery of EPCs following the establishment of CKD resulted in significant improvements in indirect measures of kidney function, with reductions in fibrosis, inflammation, and necrosis in glomerular and peritubular regions histologically.^87,88^ Chade et al have reproduced these findings in a porcine model of CKD with atherosclerosis and unilateral renal artery stenosis.^89,90^ Interestingly, in these chronic disease models, improved kidney function is not noted until 10 or more weeks after treatment, suggesting that the single dose provides lasting effects by initiating a reparative process to reverse the pre-existing kidney dysfunction. Most closely modeling human disease, EPCs have been applied in diabetic rodents with DKD.^91–93^ In models of streptozotocin-induced hypoinsulinemia or spontaneous hyperglycemia, acute intravascular or intracapsular EPC treatment results in significant improvement in measures of kidney function, with corresponding histological improvements.^91–93^ Though the kidney dysfunction in these studies was not fully corrected with treatment, they highlight the potential of EPC therapy to significantly improve established kidney disease, an important consideration for their clinical application in patients with pre-existing DKD.

Testing the concept that EPCs could be effective in diseases with a common underlying endothelial pathology, Yuen et al investigated the ability of EPCs to correct both cardiac and kidney disease resulting from severe kidney disease.^94,95^ Here, delivery of rodent EPCs resulted in reductions in fibrosis of both myocardial and glomerular tissue, while significantly improving urine PCR and diastolic cardiac dysfunction. Importantly, these investigations highlight the link between cardiac and kidney disease as it progresses clinically, and the potential for EPC therapy to significantly mitigate such end organ dysfunction in both systems.

Overall, significant evidence exists for the effectiveness of EPC treatment in preclinical models of cardiac and kidney disease, with demonstrable benefit in diabetic models of these diseases. In total, these investigations provide ample preclinical evidence for the potential clinical efficacy of EPCs.

## Clinical studies of EPCs for cardiac and kidney disease

While much focus in the past 2 decades has been directed toward clinical studies of progenitor cell therapies, few have used protocols that reflect the promising preclinical findings of EPC therapy. Most studies did not use cell culture to select for EPCs from MNCs following isolation but used unselected or CD34 + cells isolated from bone marrow or peripheral blood. Unsurprisingly, given the heterogeneity of cell products developed and tested, a variety of results have been obtained for both acute^96–98^ and chronic cardiac disease,^99–104^ with moderate to no beneficial effects found.

In contrast, beneficial effects have been seen in the few studies employing culture-derived EPCs. The TOPCARE-AMI trial^105^ demonstrated the efficacy of intra-coronary delivery of EPCs in the post-myocardial infarction period. While this trial was powered for safety and feasibility, benefits in LV ejection fraction were found in comparison to a non-randomized matched reference group. The beneficial effects of such EPCs were also found in patients with chronic total coronary artery occlusion. In a randomized controlled trial by Erbs et al,^106^ peripheral blood derived EPCs were delivered via intra-coronary infusion, yielding significant improvements in LV ejection fraction and coronary flow reserve. Though not designed to assess diabetic cardiac disease, roughly a quarter of the subjects in these studies were diabetic, suggesting the efficacy of this therapy for diabetic cardiac disease. Such positive effects, although moderate, in both acute and chronic cardiac disease highlight the potential of EPC therapy for cardiac disease.

There is no clinical-level evidence for the therapeutic potential of EPCs in kidney disease. Cellular therapy for kidney disease is still in the early stages of development with existing investigations demonstrating the feasibility of progenitor cell therapies for CKD.^107^ Accordingly, the optimal cellular therapy for CKD, and certainly DKD, remains to be determined.

Importantly, while the aforementioned studies included diabetic patients, the performance of EPC therapy in diabetic cardiac and kidney disease specifically, remains unknown. Studies focusing on EPC therapy reflective of preclinical approach for these connected entities are currently lacking, in part due to the challenges that have emerged in cellular therapy.

## Challenges and future directions

Despite the promising preclinical results of EPC therapy for cardiac and kidney disease, their translation into clinical practice has yet to be realized. As discussed, the results of trials employing EPCs, or other progenitor cells for cardiac disorders, have shown only modest benefit compared to the preclinical investigations.

### Challenges in EPC therapy for diabetic cardiac and kidney disease

Such shortcomings underscore the complexity of clinically translating EPC therapy, which requires the weighing of various factors while addressing the inherent challenges.

The first consideration is the cell donor. While some studies used allogenic cells, the majority have focused on autologous cells, hoping to prevent rejection and maximize the therapeutic effect. However, in clinical studies, autologous cells are derived from patients with pre-existing disease (ie, CVD, DM) which is known to attenuate the regenerative capacity of EPCs.^66,67,71-73^ Additionally, autologous cell therapy requires cell isolation and processing to be done prior to their use, which is inefficient in comparison to a pre-prepared allogenic source.

A secondary consideration is the source of progenitor cells, with a decision to be made between bone marrow or peripheral blood. While most studies have used peripheral blood derived-cells due to the ease of access, this source contains fewer, and often more mature, progenitor cells than bone marrow. Thus, a limit on cell number and potential potency is generated with a standard peripheral blood source.^108^

Processing following isolation is also a significant factor. Indeed, most clinical studies did not employ a cultured EPC population, but rather, a selected progenitor cell one. While this is more efficient and provides a purer product, it does not mimic the preclinical work and may contribute to the diminished potency in those studies.

Moreover, the optimal route for delivery of progenitor cells remains in question. Intravascular delivery is less invasive and has been shown to be effective, with EPCs homing to sites of injury.^77,78^ In comparison to direct tissue delivery, however, cell retention and potency maybe reduced. Whether intra-parenchymal or renal vascular delivery may be more effective in kidney disease remains to be determined. Further, whether systemic therapy could provide benefits when targeting multiple organs has yet to be assessed.

Finally, identifying the optimal patient population and therapeutic timing remains a difficult balance. The relative effect of therapy in early-stage disease is generally minimal, whereas treatment in the later stages is futile due to advanced disease.^109^ This issue is compounded in kidney and cardiac disease as patients with advanced disease have fibrosis and scar tissue that may hinder EPC penetration of the respective tissues.

Thus, the complexities of EPC therapy are many, with trade-offs to be made, and significant challenges yet to be addressed.

### Future directions

Amongst the major issues for EPC therapy is the noted dysfunction of autologous cells derived from patients with pre-existing disease.^66,71-73^ This difference from preclinical studies may explain the blunted therapeutic effects observed in clinical investigations. However, if autologous EPCs could be enhanced, their regenerative capacity could potentially be restored.

Notably, ample preclinical evidence exists for EPC enhancement by preconditioning with potent signaling molecules or through genetic manipulation.^110^ Among these approaches, modification of endothelial nitric oxide synthase (eNOS) expression is one of the most promising. eNOS is an ideal target for the rescue of EPC dysfunction as eNOS activity and NO availability are significantly reduced in many disease states, including diabetes.^31,111–113^ Moreover, NO is a key mediator of the regenerative effects of EPCs.^114^ Thus, increasing EPC eNOS expression and activity may restore their full regenerative capacity. Indeed, such modifications have proven beneficial in preclinical models of pulmonary^115^ and cardiac disease.^116^

Extending from the work employing eNOS transfected EPCs for models of pulmonary disease and cardiac disease, our group is testing the effectiveness of human EPCs with and without eNOS transfection in a xenogenic model of kidney injury using immune deficient non-obese diabetic (NOD/SCID) mice. By employing human cells in this model, we aim to inform future clinical studies employing this approach. Similarly, based on the potency of eNOS transfection in porcine models of myocardial infarction,^116^ the ENACT-AMI trial (NCT00936819)^117,118^ was developed to evaluate the potential of eNOS transfected EPCs to ameliorate cardiac function following myocardial infarction. While the publication of these studies is forthcoming, initial results (provided by personal communication) appear promising and will provide insight into the efficacy of this approach for progenitor cell augmentation in the management of kidney and cardiac disease.

Alternatively, some groups have moved away from cell therapy considering the evidence that EPCs exert their regenerative effects via paracrine mechanisms. Such effects are mediated by the EPC secretome, which consists of various stimulatory and regenerative molecules, including growth factors, cytokines, and extracellular vesicles. Extracellular vesicles (EV) are of particular interest given their ability to effect target cells via delivery of mRNA, miRNA, and proteins.

Indeed, promising results have been demonstrated by EPC-derived EVs (EPC-EV) in pre-clinical applications.^119^ EPC-EVs exert direct effects on endothelial cells, protecting them from oxidative stress and stimulating local endothelial cell repair of the vascular endothelium.^120–122^ Similarly, when applied to a rodent model of diabetic vascular disease, EPC-derived exosomes improved vascular responsiveness and reduced atherosclerosis.^123^ In models of cardiac disease, EPC-EVs demonstrate protective effects following myocardial infarction,^124^ with equivalent efficacy to EPC therapy when delivered via hydrogel to post-ischemic myocardium in rats.^125^ Likewise, EPC-EVs have significant protective effects in models of ischemic kidney injury, improving kidney function, inflammation, and resident cell apoptosis via effector miRNAs.^126–128^

Like EPCs, the composition of EPC-EVs can be modified by culture conditions or cell transfection to increase their potency.^119^ However, EPC-EVs possess some distinct advantages over cellular therapy. These include low immunogenicity, allowing for potential universal donation,^127,129^ and the possibility of de novo synthesis of EV-mimetics, allowing for efficient production.^130^ Thus, while still in the early stages, with optimal delivery and therapeutic durability to be determined, future therapies may be progenitor cell-derived rather than cell-based.

**Figure 2.**
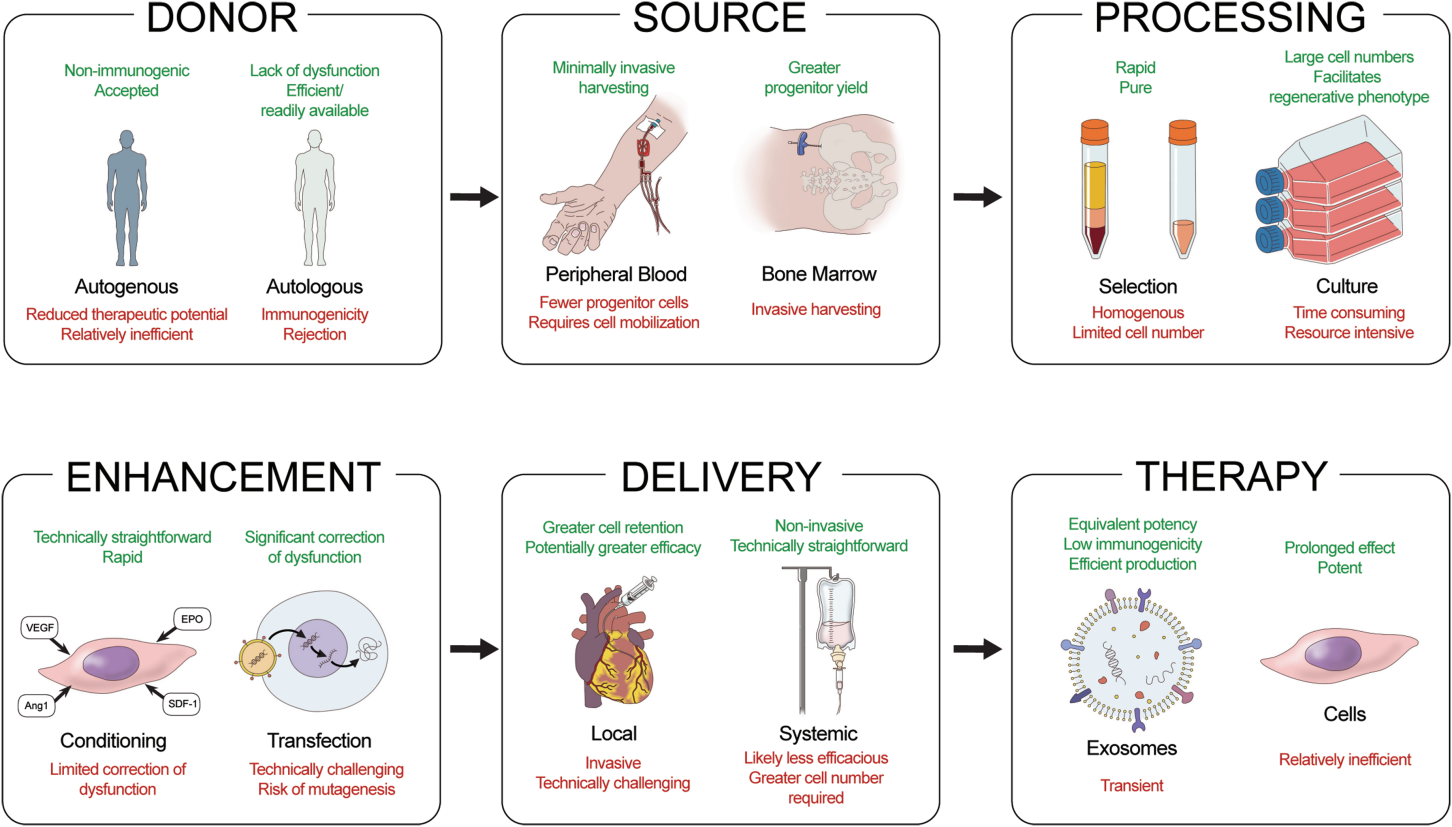
Diagram summarizing the approaches for endothelial progenitor cell-based therapy. Strengths (top) and weaknesses (bottom) of each approach relative to its alternative at key decision points in treatment development are depicted. Abbreviations: VEGF, vascular endothelial growth factor, EPO, erythropoietin, SDF-1, stromal cell-derived factor 1, Ang1, angiopoietin 1.

Overall then, while the application of EPC therapy for diabetic cardiac and kidney disease remains challenging, novel strategies with the potential to address these issues are being developed and tested (see Figure 2 for a summary of therapeutic approaches).

## Conclusion

In summary, the twin pathologies of diabetic cardiac and kidney disease are manifestations of the underlying endothelial dysfunction inherent in diabetes mellitus. Diabetes negatively impacts the intrinsic endothelial repair mechanisms, potentiating endothelial dysfunction, and necessitates extrinsic therapies to address the disorder. Extensive preclinical studies have demonstrated the efficacy of EPCs to alter this dysfunction in cardiac and kidney disease models, with few clinical studies applying similar cellular products for the treatment of cardiac disease. Current and future investigations will outline their efficacy in diabetic cardiac and kidney disease while investigating the potential for EPC or EPC-EV enhancement to augment their regenerative capacity. Thus, EPCs represent an important potential therapy for the significant clinical challenge of diabetic cardiac and kidney disease.

## Data Availability

No new data were generated or analyzed in support of this research.
